# Identification of autophagy-associated genes and prognostic implications in adults with acute myeloid leukemia by integrated bioinformatics analysis

**DOI:** 10.3389/fonc.2022.1074057

**Published:** 2023-01-13

**Authors:** Jing Zhang, Ying-Jun Wang, Yan-Qiu Han

**Affiliations:** ^1^ Department of Hematology, the Affiliated Hospital of Inner Mongolia Medical University, Hohhot, China; ^2^ National Clinical Research Center for Hematologic diseases, the First Affiliated Hospital of Soochow University, Suzhou, China; ^3^ Department of Laboratory Medicine, the Affiliated Hospital of Inner Mongolia Medical University, Hohhot, China

**Keywords:** acute myeloid leukemia, autophagy, prognosis, biomarker, bioinforamtics

## Abstract

Acute myeloid leukemia (AML) is one of the most common malignant blood neoplasma in adults. The prominent disease heterogeneity makes it challenging to foresee patient survival. Autophagy, a highly conserved degradative process, played indispensable and context-dependent roles in AML. However, it remains elusive whether autophagy-associated stratification could accurately predict prognosis of AML patients. Here, we developed a prognostic model based on autophagy-associated genes, and constructed scoring systems that help to predicte the survival of AML patients in both TCGA data and independent AML cohorts. The Nomogram model also confirmed the autophagy-associated model by showing the high concordance between observed and predicted survivals. Additionally, pathway enrichment analysis and protein-protein interaction network unveiled functional signaling pathways that were associated with autophagy. Altogether, we constructed the autophagy-associated prognostic model that might be likely to predict outcome for AML patients, providing insights into the biological risk stratification strategies and potential therapeutic targets.

## Introduction

Acute myeloid leukemia (AML), the most common acute leukemia in adults, is a heterogeneous group of leukemias characterized by aberrant clone transformation of hematopoietic precursors through the acquisition of chromosomal arrangements and abnormal gene expression patterns, exhibiting partial or complete arrest of maturation in the bone marrow, peripheral blood or other tissues (1). With application and refinement of the detection methodology such as chromosome banding, fluorescence *in situ* hybridization/chromosomal painting and the next generation sequencing, there have been incremental understanding of abnormal genetic and molecular alterations in the pathogenesis of AML (2).With these efforts, it is gradually accepted that AML is rather an umbrella diagnosis that comprises diverse subtypes with different prognostic and predictive markers, which are recommended for distinguished classification criteria and require selective and possible targeted therapies (3, 4). However, approximately half of AML patients lack predicable or prognostic biomarker and widely variable transcriptome data and the overall prognosis remains dismal [5-year overall survival 28.7%] (5), highlighting the need for identifying novel genetic and molecular predictors.

Autophagy is a critical intracellular degradative process, leading to the turnover of cellular material and providing macromolecular precursors (6). Dysfunctional autophagy has been implicated linked to multiple disorders especially in cancer cells (7), but its biological roles vary a lot and the pathophysiologic mechanism has not yet been fully elucidated. As is mentioned, autophagy is required for hematopoietic stem cell (HSC) survival as well as normal hematopoiesis (8). Dysfunctional autophagy raises the occurrence of hematological malignant neoplasms especially in leukemia (9). However, the exact role of autophagy in leukemogenesis remains debatable, in that it appears to be both leukemia-promoting and -suppressive. Some thought that reduced level of autophagy-related genes might be beneficial for AML cells due to decreased autophagic flux with accumulation of impaired mitochondrial within leukemic cells. For example, a body of evidence suggested that key autophagy genes such as *ULK1*, *ATG3*, *ATG4D* and *ATG5* were significantly downregulated in primary AML cells compared to normal granulocytes (10). Lower expression of *Beclin-1*, *LC3*, *UVRAG*, *Rubicon* and *NBR1* were identified in the high-risk AML patient group with higher white blood cell(WBC) counts and worse overall survival (11). Marine studies demonstrated that inhibition of autophagy by deletion of *Atg5* or *Atg7* prolonged survival in leukemic mice and decreased functional leukemia-initial cells (LICs) (12). However, others found that the autophagy flux was significantly higher in AML patients with TP53 mutations and inactivation of the autophagy triggered a p53-dependent increase in apoptosis in AML CD34^+^ cells (13). Nguye et al. reported loss of the autophagy receptor p62 deteriorated the expansion and colony-forming ability and impaired leukemia progression in murine models (14). Moreover, autophagy could lower the risk of myelodysplastic syndromes (MDS) progression to AML by suppress ROS levels (15).

Given the above, it is reasonable to believe that autophagy participates in the initiation and progression of AML due to its diverse roles. A deep understanding of autophagy in AML might contribute to identifying novel biomarkers in terms of diagnosis, risk stratification, prognosis as well as potential therapeutic targets. Given the functional role and therapeutic potential of autophagy in AML, we screened 12 key genes from autophagy-associated genes, and constructed risk scores that significantly predicted survival outcomes of AML patients in the study. The scoring system was also validated in an independent AML cohort. Moreover, we explored the biological pathways in that autophagy-associated genes are mainly involved. Overall, our study illustrates that the autophagy-associated model which might provide previously unrecognized risk stratification options for AML patients, shedding novel insights on potential personalized therapeutic strategies.

## Methods

### Selecting autophagy-associated signature

Autophagy-associated genes were collected by retrieving the GeneCards website (https://www.genecards.org/ ) using the term “autophagy”. Relevance scores denote the correlation between autophagy activity and individual genes. A total of 117 autophagy-associated genes were identified with relevance score at the criteria of |logFC | ≥ 1 and P-value< 0.05.

### Data collection

The gene expression matrix and corresponding clinical parameters of AML patients were collected from TCGA database, consisting of 200 adult patients and 51normal controls. The validation cohorts were downloaded from Gene Expression Omnibus (GEO, https://www.ncbi.nlm.nih.gov/geo/), under the accession of GSE23143 (16).

### Hierarchical clustering analysis and principal component analysis

Hierarchical clustering analysis of the Euclidean distance of all collected autophagy-associated genes was used to investigate the subtypes of AML. The discrimination and accuracy of the subtypes of AML patients were further evaluated by principle component analysis (PCA).

### Construction of prognostic model based on autophagy-associated genes

Differential expression analyses were used to filter autophagy-associated genes that were also significantly differentially expressed between the high-risk groups and low-risk groups. Count matrices were loaded in R package “limma” (version 3.38)[PMID: 28367255]. Significant genes were selected at the criteria of |log2 fold change | > 1 and false discovery rate (FDR)< 0.05. In total, 6061 genes were kept for the following analyses.

The optimal autophagy-associated prognostic model was constructed by multivariable Cox regression method with the least absolute shrinkage and selection operator (LASSO) algorithm in the R package “glmnet” (version 2.0-18) (17). Using the 10-fold cross-validation, the best lambda that achieved the best model performance was selected. The risk score was calculated using the formula below.


$$risk\;score=\sum_{j=1}^{n}coef\;\left(j\right)*Expr\left(j\right)$$

Coef(j) denotes the coefficient of j gene in the Cox model, and Expr(j) represents the expression levels of autophagy-related gene j. The median risk score was selected as the cutoff to separate AML patients into high-risk and low-risk groups. The same method was applied in another independent AML cohort (GSE23143), to further evaluate the predictive power of the Cox model that was trained in TCGA data.

The time-dependent receiver operating characteristic curve (ROC) was used to estimate the sensitivity and specificity in the R package “survival ROC” (version 1.0.3) (18). The area under the curve (AUCs) estimated the prognostic accuracy for 1-, 3-, and 5-year overall survival respectively, to evaluate the predictive power of survival prediction using the selected 12 autophagy-related genes. Kaplan-Meier survival curve analysis was performed and visualized by R package “survival” (version 3.1-12). Log-rank test was applied to check the significant differences between high-risk and low-risk groups. Multivariate Cox regression and univariate Cox regression were applied to investigate the associations between genes within the 12 genes and overall survival.

### Pathway enrichment analysis and regulatory network

Pathway enrichment analyses in Gene Ontology (GO) databases, including biological process, cellular component, and molecular function, were performed and visualized in the R package “clusterProfiler” (version 3.10.1) (19). These analyses were also conducted in the Kyoto Encyclopedia of Genes and Genomes (KEGG) database (https://www.genome.jp/kegg/ ). Significant pathways were identified as these with adjusted p values less than 0.05.

Protein-protein interaction networks were built and visualized on STRING website (https://string-db.org/ ), with all autophagy-associated genes.

### Construction of nomogram model

Individual genes within 12 selected autophagy-associated genes were used to build a nomogram, using the R package “survival” and “rms” (version 6.0-1). Calibration curves were plotted to evaluate the concordance between actual survival and predicted survival for 6 months, 1 year, and 3 years. The concordance index (C-index) was used to measure the model performance for predicting prognosis.

### Statistical analysis

All the statistics were conducted in the R software (version 3.5.2). The Wilcoxon test was applied to compare two groups with nonnormally distributed data. The Kruskal-Wallis tests were used for comparing more than two groups with nonnormally distributed data. Correlation coefficients were assessed by Spearman or Pearson correlations. Statistical significance in survival analysis was determined by the log-rank test. Significant p values were denoted as follows: ns ≥0.05, *<0.05, **<0.01, ***<0.001, and ****<0.0001. The statistical information for the experiments is detailed in the figure legends.

## Results

### Identification of prognostic signatures from autophagy-associated genes in AML

**Figure 1:**
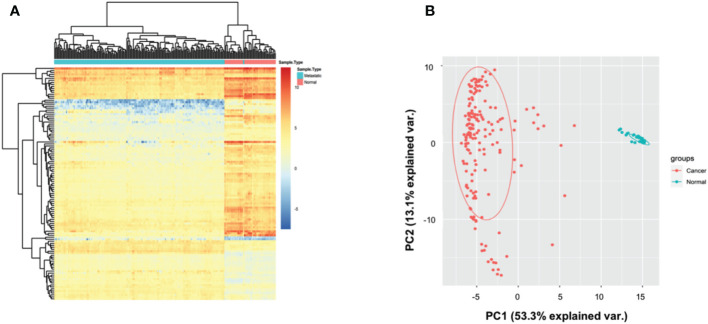
Autophagy-associated genes distinguished AML patients from controls. **(A)** Heatmap showing that the hieratical clustering of autophagy-associated genes separated AML patients from normal controls. **(B)** Scatterplot showing the gene expressions of autophagy-associated genes in AML patients are different from normal controls by PCA analysis.

A total of 200 AML patients and 51 matched healthy controls (HC) were collected in TCGA database, and the autophagy-related genes were retrieved from Genotype-Tissue Expression database. Based on hieratical clustering of gene expressions of autophagy-related genes, the AML patients and HCs were divided into two different clusters (**Figure 1A**). Consistently, the principle component analysis also indicated that AML patients were separated from HCs clusters (**Figure 1B**). These results suggested that autophagy-related genes expressed differently between AML patients and HCs in general.

**Figure 2:**
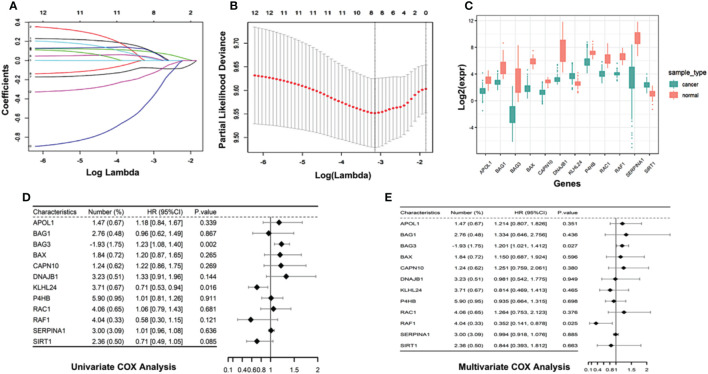
Construction of autophagy-associated prognostic model in TCGA-AML cohort. **(A)** 10-fold cross-validation of tuning parameter selection in LASSO model using the TCGA- AML data. **(B)** LASSO coefficient of the selected 12 autophagy-associated genes. **(C)** Boxplot comparing the difference in gene expression of the selected 12 autophagy-associated genes between AML patients and normal controls. **(D, E)** Univariate Cox analysis **(D)** and multivariate Cox analysis **(E)** show the coefficients and corresponding p values of individual genes within 12 autophagy-associated genes.

To recognize the prognosis-related genes from the autophagy-associated genes, we identified 117 differentially expressed genes (DEGs) between AML patients and normal healthy controls, and then performed Cox regression model with LASSO algorithm. Using the best lambda parameters in the 10-fold validations, we finally selected 12 hub autophagy-associated genes, including *APOL1*, *BAG1*, *BAG3*, *BAX*, *CAPN10*, *DNAJB2*, *KLHL24*, *P4HB*, *RAC1*, *RAF1*, *SERPINA1*, and *SIRT1* (**Figures 2A, B**), which, as expected, were significantly differentially expressed in AML patients (**Figure 2C**). Univariate Cox analysis revealed that some of the 12 genes were correlated to the overall survival (OS) of AML patients. For instance, *KLHL24* has a hazard ratio of less than 1 in AML patients and was regarded as a protective gene (HR=0.71, 95% CI=0.53-0.94, P=0.016, **Figure 2D**), while *BAG3* was considered a risk gene (HR=1.23, 95% CI=1.08-1.40, P=0.002, **Figure 2D**). Multivariate Cox analyses were also conducted to confirm that *BAG3* was significantly correlated with worse overall survivals of AML patients (HR=1.201, 95% CI=1.021-1.412, P=0.027, **Figure 2E**). These analyses are consistent with the indispensable role of *BAG3* in cancer progression and tumor resistance to therapy (20).

### Reconstruction of prognostic evaluations for AML patients

**Figure 3:**
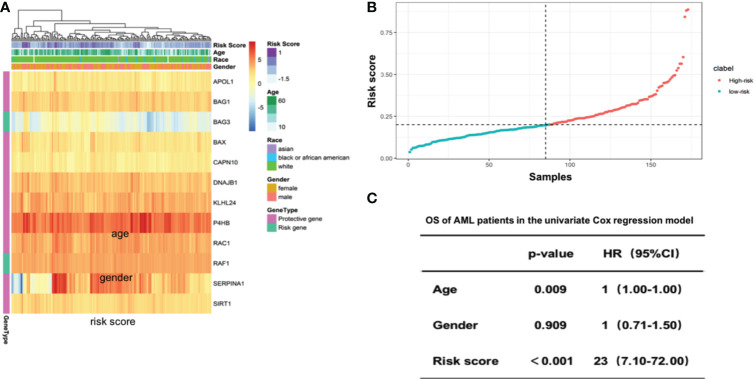
Risk score of 12 selected autophagy-associated genes is an independent predictor of patient survival in the AML cohort. **(A)** Heatmap showing the gene expression of the 12 selected autophagy-associated genes. Columns were annotated by patient race, gender and age. **(B)** Rank of risk scores in AML patients who were divided into high-risk and low-risk groups by the median value of risk score. **(C)** Multivariate Cox analysis shows the coefficients and corresponding p values in the model with risk score, age and gender as covariates.

We then calculated the risk score for individual AML patients based on the gene expression patterns and coefficients in LASSO model. Specifically, the risk score was the sum of gene expressions of the 12 autophagy-associated genes weighted by their corresponding coefficients in multivariable LASSO regression (Methods). The unsupervised hieratical clustering of gene expression profiles of 12 autophagy-associated genes exhibited that their expressions and the corresponding risk scores were confounded by age, gender and race in AML (**Figure 3A**). AML patients were then divided into the high-risk and low-risk groups based on the median value of risk scores (**Figure 3B**). Next, we evaluated the predictive power of the risk score in prognosis of AML patients. Compared with age and gender, risk scores indicated the highest hazard ratio with the OS of AML patients in the univariate COX regression model (HR=23, 95% CI=7.1-72, P<0.001, **Figure 3C**). These results revealed that the risk scores were an independent prognostic predictor in AML.

### Evaluation of the prognostic model in AML

**Figure 4:**
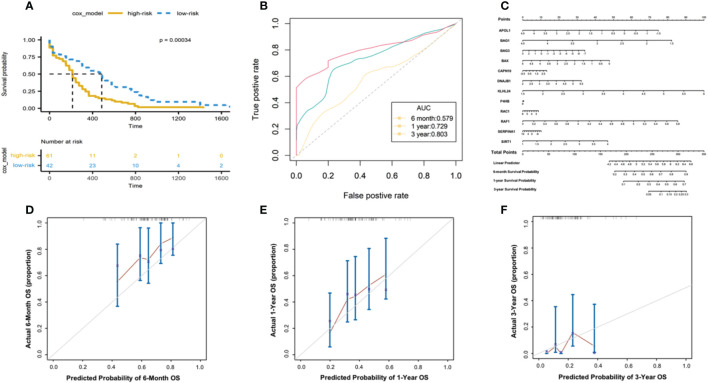
Evaluating the predictive power of risk scores in TCGA-AML cohorts. **(A)** Kaplan-Meier analysis based on the 12-gene signature in TCGA- AML cohort. Color denotes patients’ stratifications based on risk scores. The table below shows the alive patients at individual time points. **(B)** The ROC curve showing the AUC of the predictions for 6-month, 1-year and 3-year survivals. **(C)** Nomogram constructed between the 12 autophagy-associated genes and 6-month, 1-year and 3-year survivals. The calibration plot of the nomogram showing the observed survivals and Nomogram-predicted survivals for 6-month **(D)**, 1-year **(E)** and 3-year **(F)** survivals.

To evaluate the predictive power of the risk score in AML prognosis, we conducted Kaplan-Meier analyses, and found the AML patients with high-risk scores showed significantly worse overall survivals than patients with relatively low-risk scores in TCGA database (**Figure 4A**). The time-dependent ROC curve also revealed that the risk scores were capable to predict 6-month (**Figure 4D**), 1-year (**Figure 4E**) and 3-year (**Figure 4F**) survivals with area under curve more than 0.579 (AUC for 6 months, 0.579; AUC for 1 year, 0.729; AUC for 3 years, 0.803, **Figure 4B**).

Next, we developed a nomogram model of individual autophagy-associated genes to predict patients’ survivals (**Figure 4C**). The calibration curve showed that the combination of these autophagy genes achieved better performance for AML patients’ 6-month, 1-year prognosis, compared with each one alone (**Figure 4D**). These findings consistently revealed the success of 12 autophagy-related genes in predicting patient survivals, suggesting the critical role of autophagy signaling in the development of AML progression.

### Validating the performance of the autophagy-associated prognostic model

**Figure 5:**
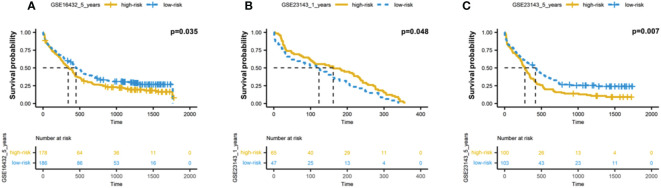
Validation of autophagy-associated prognostic model in independent AML cohort. **(A-C)** Kaplan-Meier analysis of 6-month **(A)**, 1-year **(B)** and 3-year **(C)** survivals based on the 12 autophagy-associated genes in GSE23143 cohort.

We further validated the prognostic model in independent dataset (GSE23143), including the 200 AML patients. Applied the same methods, we calculated the risk scores based on the 12 autophagy-associated genes, and then divided AML patients into two groups, including high-risk and low-risk groups. Consistent with the results in TCGA data (**Figure 4A**), we found the high-risk groups showed the significantly worse overall survivals for 6 months, 1 year, and 3 years (p-value = 0.035 for 6 months; p-value = 0.048 for 1 year; p-value = 0.007 for 3 years; **Figures 5A-C**). These results suggested the effectiveness of autophagy-associated model in predicting prognosis in AML cohorts.

### Pathway analyses showed the autophagy-related signaling pathways

**Figure 6:**
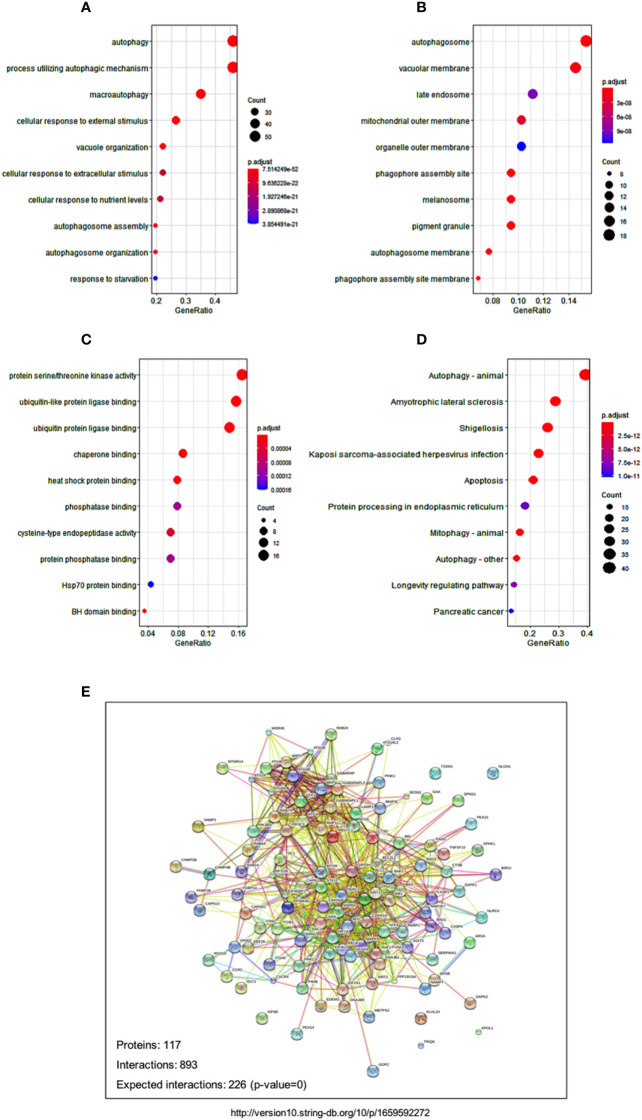
Signaling and networks related to autophagy-associated genes. **(A-C)** Dot plot reveals the GO enrichment of autophagy-associated genes, showing the significant biological pathways **(A)**, cellular components **(B)**, and molecular functions **(C)**, respectively. **(D)** Dot plot displays the significant pathways that were enriched in KEGG databases. Dot sizes denote the count number of enriched genes in the pathway, and dot color represents the adjusted p value of the enriched pathway. **(E)** Protein-protein interaction network shows the relationships among autophagy-associated proteins in the STRING database.

In order to decipher the biological pathways related to autophagy-related risks, we viewed the 12 autophagy-associated genes as baits to find 106 most related neighbor genes in TCGA-AML cohort. Totally, 106 genes were identified as neighbor genes. Biological pathway analysis in GO database showed that cellular response to nutrients levels and response to starvations pathways were prominently related to the 12 genes, in addition to autophagy pathway (**Figure 6A**). Cell component analyses in GO databases showed consistently enriched in the autophagosome and autophagosome membrane (**Figure 6B**). Molecular function enrichment displayed several related functions, such as protein serine/threonine kinase activity, ubiquitin-like protein ligase binding and ubiquitin protein ligase binding (**Figure 6C**). These findings are also in line with the fact that the ubiquitin-proteasome system and autophagy are two major quality control systems responsible for protein degradation (21).

Pathway enrichment in KEGG database showed several disease-related pathways, including amyotrophic lateral sclerosis, shigellosis, and Kaposi sarcoma-associated herpesvirus infection (**Figure 6D**). The significant enrichments of disease pathways indicated that the 12 autophagy-associated genes might play an essential role in the development of these human diseases. The tight protein-protein interaction network of the 12 autophagy genes and their neighbor genes reflect their close relationships as well (**Figure 6E**).

## Discussion

The genetic alterations in AML are highly heterogeneous and the manifestation of the disease is distinguishing in each patient. New therapeutic targeted drugs have shown promising effects but these are directed only to specific AML subgroups (22). There still remains around half of the patients who cannot be reasonably categorized due to a lack of prognostic biomarkers. Given this context, there is urgent need to better understand the molecular mechanisms pathogenesis involved in AML and novel classification systems are needed to improve the accuracy of predicting patients’ prognosis.

In this study, we focused on the autophagy-associated genes due to their essential roles in leukemogenesis. We constructed a multivariate prognostic model and identified 12 key autophagy associated genes that indicated significant relevance with prognosis. The filtered 12 genes provided previously unrecognized stratification strategies for AML patients, and also potentially promising targets for AML treatments (23, 24).

We constructed the autophagy-based risk scores based on 12 LASSO regression-selected prognostic genes. The scoring systems are robust to predict the outcome of AML patients in both TCGA-AML cohorts and another independent cohort. Multivariate Cox analysis also revealed that the risk scores were an independent factor, but not the age and gender. These findings are consistent with previous studies that have reported autophagy was involved in cancer initiation *via* regulating many oncogenes and tumor suppressor genes (25–27). Altogether, our analysis suggested the undermined role of autophagy in AML development.

Among the selected 12 key genes, we identified several genes which have been reported to participate in tumor prognosis and tumorigenesis. For instance, Bcl2-associated athanogene-3 (BAG3), also known as CAIR-1(CAI-stressed-1), belongs to the family of co-chaperones interacted with the ATPase domain of the hear shock protein Hsp 70 *via* the structural domain known as BAG domain(110-124 amino acids) (28). *BAG3* gene expression is constitutive in normal cells such as the skeletal and heart muscles, while aberrant expressed *BAG3* is also found in neoplastic cell lines as well as primary AML and CML cell (29, 30). Studies has confirmed that overexpressed *BAG3* could reverse the pro-apoptotic effect of WT1 silencing and regulate the leukemia stem cell-supporting activity (31). Further evidence demonstrated that *BAG3* down-modulation resulted in a reduction of the anti-apoptotic protein level such as MCL1, BCL2 and BCL-XL, which are capable of regulating autophagy in AML cells (32). It is expected that *BAG3* will serve as a key player in leukemogenesis and potential therapeutic drug target (33–35). RAC1, a member of the Ras superfamily of small guanosine triphosphatases (GTPases), is capable to activate several signaling pathways and cytoskeletal arrangements, resulting in cell cycle progression, morphogenesis, migration as well as autophagy (36). Abnormal overexpression has been regularly reported in cancer. Early research indicated that inactivation of RAC1-GTPase suppressed migration and promoted drug induced apoptosis in KG-1 cells (37). Recent *in vitro* studies found that suppression of RAC with a RAC inhibitor (EHT-1864) could increase autophagy, apoptosis, cell cycle, modulation of p53 factor and inhibit the PI3K/AKT/mTOR signaling pathway in AML cell lines (38). In fact, it has recently been reported that the combination of EHT-1846, venetoclax(BCL-2 inhibitor) and midostaurin(FLT3 inhibitor) could reverse midostaurin resistance in AML cells (39). Sirtuin 1, known as NAD-dependent deacetylase sirtuin-1, promoted cancer cell proliferation and metastasis *via* STAT3/MMP-13 signaling (40), which is also found participated in the abnormal metabolism pathways in AML. Recent study suggested that SIRT1 was a downstream factor of AdipoR1 and ANRIL in glucose metabolism and regulate AML cell survival (41). Apolipoprotein L1 (APOL1) functions as both extra- and intra-cellular regulators in host innate immunity and cellular homeostasis in the kidney (42). It is worth noting that these identified autophagy genes have not been fully recognized in the development of hematopoietic disorders especially in AML, which might provide novel promising molecular targets and help to predict the outcomes, monitor the minimal residue disease and find therapeutic targets in AML.

The protein-protein interaction analysis and pathway enrichment results demonstrate that autophagy is related to environmental stimulations. Similarly, autophagy can respond to a wide spectrum of cellular stresses, including nutrient deprivation, hypoxia, and abnormal macromolecule accumulation (43, 44). Also, KEGG enrichment analysis revealed the significant enrichment of disease-related pathways, which could be partly explained by its essential functions for cell survival, bioenergetic homeostasis, and intracellular component degradation (45). Indeed, it is gradually recognized that autophagy might be responsible for tumorgenesis in multiple direct and indirect signaling ways. For example, aberrations in metabolic rewiring has been described in leukemogenesis due to dysfunctional autophagy in recent years (46). As our analysis aimed mainly to the autophagy, it is insufficient to fully display the pathways of autophagy in leukemia pathogenesis. Additionally, in different setting of chemotherapy, immunotherapy as well as hematopoietic stem cell transplantation, the role of the identified autophagy-genes in AML still remains largely unknown and more objective proof of this waits further experimental testing and detailed functional analyses.

Besides that, there are other limitations in our study. This study aimed to identify a prognostic autophagy-associated gene signature in patients with AML. However, our analysis was mainly based on TCGA and GEO databases which could have biased our conclusions. Considering this, more validations in larger clinical population groups are required to provide more applicable results and enhance the clinical application value as prognostic tools in the AML patients.

Taken together, we recognized a 12-autophagy-associated gene signature which might likely act as an independent predictor of prognosis based on multiple AML cohorts. A nomogram model and Cox regression analyses revealed the accuracy of the gene signature in predicting 6-month, 1-year and 3-year survival probability for individual AML patients. Pathway enrichment analyses demonstrated the potentially related biological pathways of autophagy. Our finding illustrates that the 12-autophagy gene signature would provide new insight into a better understanding of autophagy in AML. Although further validation is needed, we hope it will provide promising prognostic significance and potential therapeutic targets in AML treatment.

## Data availability statement

The original contributions presented in the study are included in the article/supplementary materials. Further inquiries can be directed to the corresponding author.

## Author contributions

JZ, YQH designed the project and supervised the typescript preparation. JZ and YJW performed the analyses and interpreted all the data. JZ prepared the figures and tables. YQH reviewed and revised the manuscript. All authors contributed to the article and approved the submitted version.
